# Preliminary study of p53 and c-erbB-2 expression in gallbladder cancer in Indian patients manuscript id: 8962091628764582

**DOI:** 10.1186/1471-2407-6-126

**Published:** 2006-05-10

**Authors:** Amita Chaube, Mallika Tewari, RS Garbyal, Usha Singh, Hari S Shukla

**Affiliations:** 1 Department of Pathology, Institute of Medical Sciences, Banaras Hindu University, Varanasi -221005, India; 2 Department of Surgical Oncology, Institute of Medical Sciences, Banaras Hindu University, Varanasi -221005, India

## Abstract

**Background:**

The inactivation of the tumour suppressor gene and activation of the proto-oncogene are the key steps in the development of the human cancer. The p53 and c-erbB-2 are the best examples of it. In the present study, our aim was to determine the role of these genes in the carcinogenesis of gallbladder by immunohistochemistry.

**Methods:**

In all 78 consecutive patients of gall bladder diseases were studied for p53 and c-erbB-2 expression immunohistochemically and their expression was correlated with the age, grades and stages of the disease and presence of stone. An informed consent was obtained in each case. Chi square and z test were applied to see the association of p53 and c-erbB-2 over expression with other clinicopathological factors.

**Results:**

Eight (20%) patients of gall bladder cancer were positive for p53 expression and 10 (25%) patients for c-erbB-2. The p53 positivity increased with increasing grade while cerbB-2 positivity decreased with increasing grade of gall bladder cancer. Mean age in cerbB-2 positive cases were lesser as compared to negative cases while p53 did not show such association with age.

**Conclusion:**

Only one case of gall bladder cancer co-expressed the p53 and c-erbB-2, thereby suggesting that p53 and c-erbB-2 may have independent role in carcinogenesis of gall bladder cancer. c-erbB-2 over expression in adenoma and younger age group indicates its role as an early event in carcinogenesis of gallbladder. However study of larger sample is required to further validate the results.

## Background

Gallbladder cancer (GBC) is a latent disease, as a result it is usually diagnosed in an advanced stage, resulting a 5 year survival rate of <10% [1]. Gallbladder cancer is very common in South American countries, the Mediterranean region, Japan [2] and northern part of India [3]. Chile has the highest incidence rate of GBC (7.5/100,000 population) especially among American Indian females [4]. According to Indian Council of Medical Research cancer registry 2001 [5], in North India the incidence of GBC is 2.5–8.9/1,00,000 female and 1.6 3.7/1,00,000 male whereas in Southern India it is only 0.7–0.8/1,00,000 female and 0.50.6/1,00,000 male population. The poor prognosis and high incidence of GBC in North India necessitates a closer look at the molecular changes for evolving an effective early diagnostic and therapeutic strategy. The p53 gene, the "guardian of the genome" a tumour suppressor gene, is the basic genetic alteration in GBC [6]. A key feature of p53 is its tendency to remain "latent" in unperturbed cells. In contrast to the normal p53 gene product (protein), the mutated p53 gene product (protein) tends to accumulate in cell nuclei which can be detected by immunohistochemistry [7].

The second most common genetic change occurs is c-erbB-2, a proto-oncogene also known as neu or HER-2. HER-2 glycoprotein consist of extracellular and a cytoplasmic domain. The cytoplasmic domain has activating phosphorylation and transcription initiating function and it is the domain to which antibodies for immunohistochemical studies have been derived [8]. Alterations in cerbB-2 signalling have been implicated in neoplastic transformation of cells in vitro [9] and neoplasia in both experimental animals and human [10], however few studies have investigated the role of c-erbB-2 in human gall bladder cancer [11-14]. Over expression of c-erbB-2 indicates the worse prognosis [11,15].

A composite report on both p53 and c-erbB-2 in gallbladder cancer (GBC) in India is sparse. Therefore we have undertaken this pilot study to screen the GBC patient for both p53 and c-erbB-2 in this high prevalence area for GBC.

## Methods

The study was carried out by the Department of Surgical Oncology, Institute of Medical Sciences, Banaras Hindu University, Varanasi, India and patients from the said department formed the basis of the present research. Histopathology and immunohistochemistry of the gallbladder specimens were carried out at the UGC advanced Immunodiagnostic Training and Research Centre, Department of Pathology. The Institutes' Ethics Committee approved the study. An informed consent was obtained from each patient. Clinical details were recorded on preformed proforma in each case.

A total of 78 consecutive patients between October 2003 and May 2004, including 40 GBC, 10 adenoma, 5 carcinoma in situ, and 23 gallstone disease (GSD) were examined for p53 and c-erbB-2 immunohistochemically.

The surgically resected specimens of gallbladder cancer were fixed in 10% buffered formalin and embedded in paraffin. The cut sections were stained with haematoxylin and eosin (H&E). The diagnosis of GBC was based on clinical suspicion and histopathological confirmation in each patient. Depth of invasion and staging were judged using TNM classification by American Joint Committee in the year 2002 [16]. Histopathological grading of GBC was done according to WHO classifications [17].

The p53 and c-erbB-2 expression were examined by the immunohistochemical methods as given below-

Three to five micron thick sections were cut from the paraffin embedded tissue blocks and placed on gelatin coated slides. Immunohistochemical staining of p53 and c-erbB-2 was performed using Avidin Biotin method. Instructions of kits (Novacastra laboratories Ltd., UK.) were followed. Brief description of technique is as follows:

The sections were dewaxed in xylene and rehydrated in graded alcohol. Sections were then immersed in 1.5% H2O2 in methanol for 10 minutes followed by washing in tap water. Nonspecific binding was blocked by incubating the slide with normal rabbit sera for 10 minutes. Excess serum was shaken then sections were immediately covered with primary antibodies (ready to use) and incubated for 30 minutes in humid chamber.

The primary antibody used for p53 was NCL-p53 clone DO-7 and for c-erbB-2 detection NCL-c-erbB-2 clone CB 11 antibody. After washing in tris buffer saline (pH 7.6), secondary biotinylated antibody (rabbit anti mouse IgG with 1 in 50 dilution in TBS) was put for one hour. The slides were washed with different changes of TBS for 10 minutes. Then the slides were incubated with ABC reagent using 100 μl TBS, 1 μl avidin and 1 μl Biotin for 30 minutes. The colour was developed by using diamino benzidine (DAB) as the chromogen and slides were washed well with tap water. Finally the slides were counter stained with Mayer's haematoxylin for 2 minutes, washed, dried in alcohol and mounted with DPX mountant. Sections of carcinoma breast were taken as positive control slide for both p53 and c-erbB-2 staining.

### Criteria of positive immunohistochemical staining

The results were evaluated quantitatively as well as qualitatively according to intensity of staining pattern. According to the estimated number of positive cells they were divided into five groups [18] (- = <10 %, +1 = 10–30 % of cells positive, +2 = 30–50 % of cells positive, +3 = >50% of cells positive). The p53 and c-erbB-2 immunoreactivity were considered positive when more than 10% nuclei and membrane bound or cytoplasmic staining was observed respectively [19]. Cytoplasmic staining for p53 was disregarded. The intensity of staining was graded as weak, moderate and strong.

### Statistical analysis

Statistical analysis of the data was done on SPSS computer statistics programme. To determine the association between two or more than two variables, chi square test has been applied.

## Results

**Figure 1:**
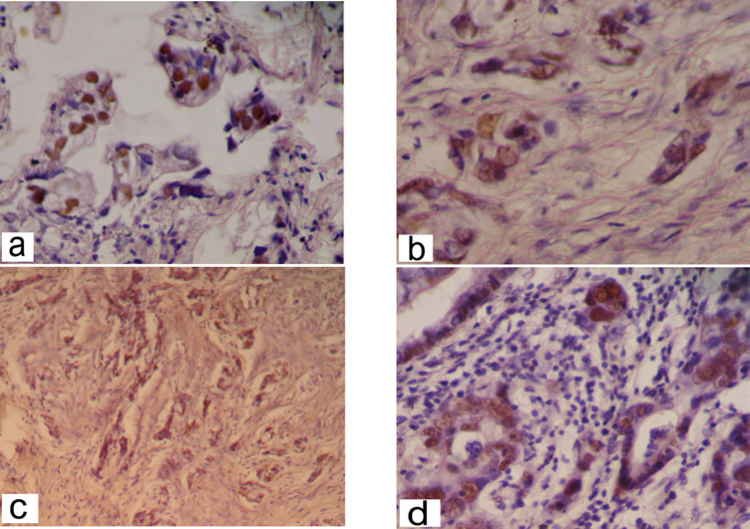
Photomicrograph showing all p53 staining. (1a) nuclear p53 positivity in well differentiated adenocarcinoma; (1b and c) in invasive high grade adenocarcinoma; (1d) strong positivity in moderately differentiated adenocarcinoma

**Figure 2:**
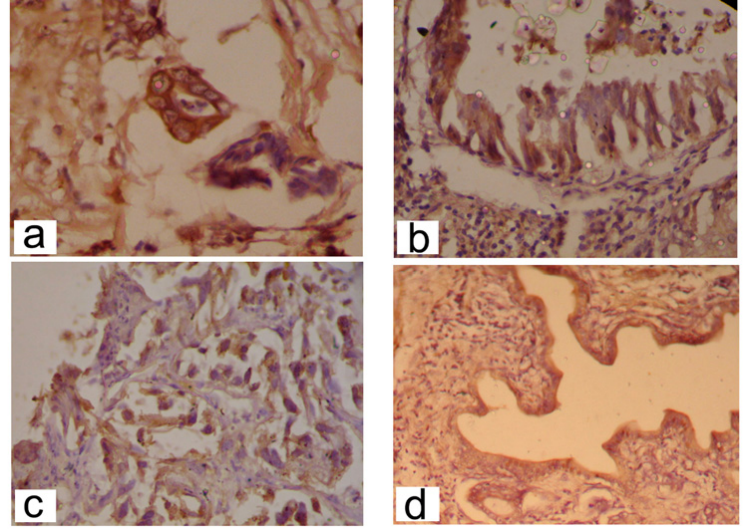
Photomicrograph showing c-erbB-2 staining. (Fig 2b and d) patchy and membranous positivity in well differentiated carcinoma; (2a) in moderately differentiated carcinoma; (2c) cytoplasmic and membranous in poorly differentiated carcinoma

**Figure 3:**
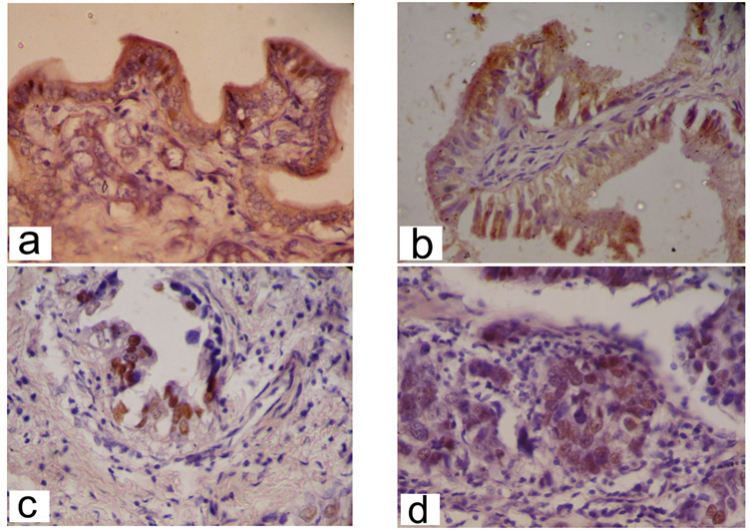
Photomicrograph showing both c-erbB-2 and p53 staining. (Figure 3a and b): apical granular and intense membranous c-erbb-2 positivity in adenoma gallbladder; (c and d): strong nuclear p53 positivity in well differentiated and poorly differentiated adenocarcinoma respectively.

The mean age of GBC patients was 53.62 ± 12.46 with age range of 30 to 70 years and female to male ratio was 3:1 while the mean age of GSD patient was 48.29 ± 14.56 years with age range of 28 to 58 years and female to male ratio was 2.5:1. The p53 over expression was found in 8 out of 40 (20%) cases of gallbladder cancer but in none of the benign diseases of gallbladder (Table 1). Amongst the patients of GBC none of the grade I cases over expressed p53, while 2/10 (20%) and 6/16 (37%) of grade II and III cases revealed over expression respectively. The immunoreactivity was mainly nuclear with only occasional cytoplasmic staining (Figure 1). Cells having invasive character showed intense nuclear p53 positivity (Figure 1b and 1c). No correlation (p = NS) of p53 positivity with the clinical stage was observed (Table 2). However there is statistically significant (p < 0.05) difference in p53 over expression pattern among grades of tumour (Table 3). Only ten out of 40 cases of GBC (25%) over expressed c-erbB-2 (Table 1). In relation to the grade of tumour 35% grade I (Figure 2b &2d), 30% of grade II (Figure 2a) and 12% of grade III (Figure 2c) GBC cases revealed c-erbB-2 over expression. Four of the 10 (40%) papillary adenoma of the gallbladder over expressed c-erbB-2 (Table 1). None of the Carcinoma in situ or GSD stained positive. Only 6 cases of GBC showed more than 40% cells with positive staining for c-erbB-2. A strong c-erbB-2 over expression was present in papillary adenocarcinoma (Figure 3a &2b). This intense staining was predominately seen in apical surfaces of the papillary epithelium and luminal surfaces of the glands. One papillary adenoma showed apical granular positivity (Figure 3b), and the second one showed intense membranous staining. In grade III adenocarcinoma cases, no membranous pattern of staining were observed, only cell clumps showed cytoplasmic staining (Figure 2c). There is no statistical difference in cerbB-2 over expression among stages of gallbladder tumour (Table 4). Likewise comparison of c-erbB-2 over expression pattern among grades of tumour revealed non significant (p = NS) difference although higher c-erbB-2 over expression was noted in low grade tumour (Table 5).

We found over expression of both p53 and c-erbB-2 in only one case in our study. There was statistically no significant difference in expression pattern of p53 and c-erbB-2 among different histological groups (p = NS) except in adenoma where only c-erbB-2 is positively expressed.

There was a significant (p < 0.05) association between p53 over expression and gallbladder cancer with stone. However c-erbB-2 over expression did not show significant association with gallstone although higher cerbB-2 positivity was noted in gallbladder cancer with associated gallstone. There was no significant correlation of p53 positivity with age and sex of the patients. Interestingly the c-erbB-2 positive cases were in mean age of 37 years as compared to 48 years for c-erbB-2 negative cases.

## Discussion

Cancer arises mainly from mutations in somatic cell. However it is not the result of a single mutation rather it results from increasing genetic disarray accumulated over the time. Therefore carcinogenesis is multistep and age dependent process [20]. The multiple mechanisms and multiple players involved in this process inspired us to evaluate the status of most commonly disrupted genes i.e. p53 and c-erbB-2 specifically the simultaneous over expression of these genes in gallbladder diseases in the same individual.

There is no literature available on p53 and c-erbB-2 study in gallbladder diseases in Indian patients except one [21]. Our region (Northern India) being the endemic to GBC, this pilot study was conducted to recognize the over expression of p53 protein and c-erbB-2 protein in gallbladder diseases. So it could be correlated with age, sex, stage, grade and lymph node status especially in GBC. This will help in developing the better therapeutic strategies for GBC.

Present study showed only 20% p53 positivity in GBC compared to the higher figures reported by other workers [15,22-24] and no p53 over expression either in premalignant condition or in GSD patients.

The role of p53 seems to be limited in the initiation of gallbladder carcinogenesis as none of the premalignant lesions and grade I GBC showed p53 over expression. The higher p53 over expression with the increasing grade of GBC suggests its role in tumour progression rather than initiation. It seems that the c-erbB-2 gene alteration is an early event and plays a critical role in gallbladder carcinogenesis as nearly 35% of grade I and 40% of adenoma showed c-erbB-2 over expression in our population. Increasing over expression of p53 with the increasing grade of the tumour and decreasing over expression of c-erbB-2 with increasing grade suggest that c-erbB-2 is likely to play a lesser role in tumour progression in comparison to the p53 gene.

We have taken all the intense c-erbB-2 membranous staining as a positive staining in well to moderately differentiated cases whereas cytoplasmic staining in few poorly differentiated cases were also regarded as a positive staining. This result is very well supported by few studies [25,26] where cytoplasmic positivity along with membranous staining was regarded as positive.

The c-erbB-2 over expression in papillary-adenoma in our results substantiates the evidence of adenoma-carcinoma sequence [27]. This is further supported by other recent studies [11,28]. In comparison to the other studies regarding the p53 and c-erbB-2 over expression percentage, our study revealed lower percentage of positivity. This disparity could be due to differences in genetic predisposition between the two populations, further study is required to establish it. There was no correlation of p53 over expression with the age and sex of the patient while c-erbB-2 was over expressed in younger age group patients, again indicating its role as an early event in carcinogenesis of gallbladder.

We have not found the co expression of c-erbB-2 and p53 in GBC except in one case of poorly differentiated carcinoma and that too showing weak positivity. This observation suggests the lack of cooperation between p53 and c-erbB-2 gene in neoplastic transformation of gallbladder. This finding is very well supported by Kim et al, 2001 [5].

## Conclusion

Both p53 and c-erbB-2 might play an independent role in gallbladder carcinogenesis. c-erbB-2 seems to play critical role in tumour initiation and p53 in the tumour progression. So cerbB-2 may be used as an important marker to identify the premalignant lesions which are likely to progress into malignancy. However further study with a big sample size is required to validate the results.

## Competing interests (financial/non-financial)

The author(s) declare that they have no competing interests.

## Authors' contributions

**AC: **Participated in data collection, conducted the experiment, performed the statistical analysis and prepared the manuscript

**MT: **Helped significantly in data collection and preparation of the manuscript.

**RSG: **Helped in the preparation of the photomicrographs and editing of the manuscript.

**US: **Performed the histopathology and immunohistochemistry and contributed in preparation of the manuscript.

**HSS: **Designed the study and edited the manuscript for its scientific content beside overall supervision of the research.

## Pre-publication history

The pre-publication history for this paper can be accessed here:



## Figures and Tables

**Table 1:** Comparison of C-erbB-2 and p53 expression in different histological Groups Z value test.

**Patient Group**		**Total Cases**	**P53 expression**		**c-erbB-2 expression**		**Z value**	**P value**
					
			**No.**	**%**	**No.**	**%**		
1.	Non-malignant gallbladder disease	23	0	0	0	0	0	
2.	Adenoma	10	0	0	4	40	2.58	<0.05
3.	Carcinoma in situ	5	0	0	0	0	0	
4.	Gall bladder cancer (Histological grade)	40	8	20	10	25	0.730	NS

**Table 2:** Correlation of p53 expression with the stage of tumour

**Patient Group**	**P53 expression**		**χ**^**2**^	**P value**
**(No. of cases)**	**(+)**	**(-)**		
Stage I (0)	0	0	0.208	NS
Stage II (8)	2	6		
Stage III (12)	2	10		
Stage IV (20)	4	16		

**Table 3:** Correlation of p 53 expression with the grade of tumour

**Patient Group**	**P53 expression**		**χ**^**2**^	**P value**
**(No. of cases)**	**(+)**	**(-)**		
Grade I (14)	0	14	6.568	0.037
Grade II (10)	2	8		
Grade III (16)	6	10		

**Table 4:** Correlation of c-erbB-2 expression with the stage of tumour

**Patient Group**	**C-erbB-2 expression**		**χ**^**2**^	**P value**
**(No. of cases)**	**(+)**	**(-)**		
Stage I (0)	0	0	1.48	0.476
Stage II (8)	2	6		
Stage III (12)	4	8		
Stage IV (20)	3	17		

**Table 5:** Correlation of c-erbB-2 expression with the grade of tumour

**Patient Group**	**C-erbB-2 expression**		**χ**^**2**^	**P value**
**(No. of cases)**	**(+)**	**(-)**		
Grade I (14)	5	9	1.75	0.416
Grade II (10)	3	7		
Grade III (16)	2	12		
